# Investigating the protective and therapeutic potential of new generation antioxidant combinations in the brain: an experimental aging model

**DOI:** 10.1007/s10522-026-10399-z

**Published:** 2026-02-05

**Authors:** Büşra Dönmez, Elif Naz Gürsoy, Kanuni Barbaros Balabanli, Şule Coşkun Cevher

**Affiliations:** 1 Department of Neurology, Ankara Bilkent City Hospital, Ankara, Turkey; 2 Department of Biology, Faculty of Science, Gazi University, Ankara, Turkey

**Keywords:** Aging, Saponin, Squalene, ROS, Foxo3a, Sirt1

## Abstract

The increasing elderly population has brought healthy aging into focus. Aging is a multifactorial process characterized by the progressive decline of cellular and tissue functions, largely due to cumulative oxidative stress. Antioxidant-based strategies have therefore gained prominence as potential interventions. This study investigated the protective and therapeutic effects of Squalene (SQ) and Saponin (SP), individually and in combination, on aging-related biomarkers in brain tissue using a D-Galactose (D-Gal)-induced rat model. Forty-eight male Sprague–Dawley rats (200–250 g) were randomly divided into eight groups (*n* = 6). Aging was induced in four groups via intraperitoneal administration of D-Gal (300 mg/kg/day) for six weeks. One group received no antioxidants, while others were treated orally with SQ (2.66 mL/kg/day), SP (100 mg/kg/day), or their combination. Non-aging groups received the same antioxidant treatments without D-Gal. At the end of the intervention, brain tissues were collected for biochemical analysis. Spectrophotometric assessments included Malondialdehyde (MDA), Glutathione (GSH), Nitric oxide derivatives (NOx), Ascorbic acid (AA), and Protein carbonyls (PC). Forkhead Box O3A (FOXO3A), Nuclear factor erythroid 2-related factor 2 (NRF2), Sirtuin 1 (SIRT1), Paraoxonase 1 (PON1), and Klotho were quantified by ELISA. Combined SQ and SP treatment significantly decreased oxidative stress markers (MDA, NOx, PC) and increased antioxidant defenses (GSH, AA) as well as aging-related molecules (FOXO3A, NRF2, SIRT1, PON1, Klotho) (p < 0.05). Serum Alanine Aminotransferase (ALT) and Aspartate Aminotransferase (AST) levels were also reduced. These findings demonstrate, for the first time, that combined SQ and SP administration can mitigate aging-related oxidative stress and molecular alterations in brain tissue.

**Graphical abstract:**

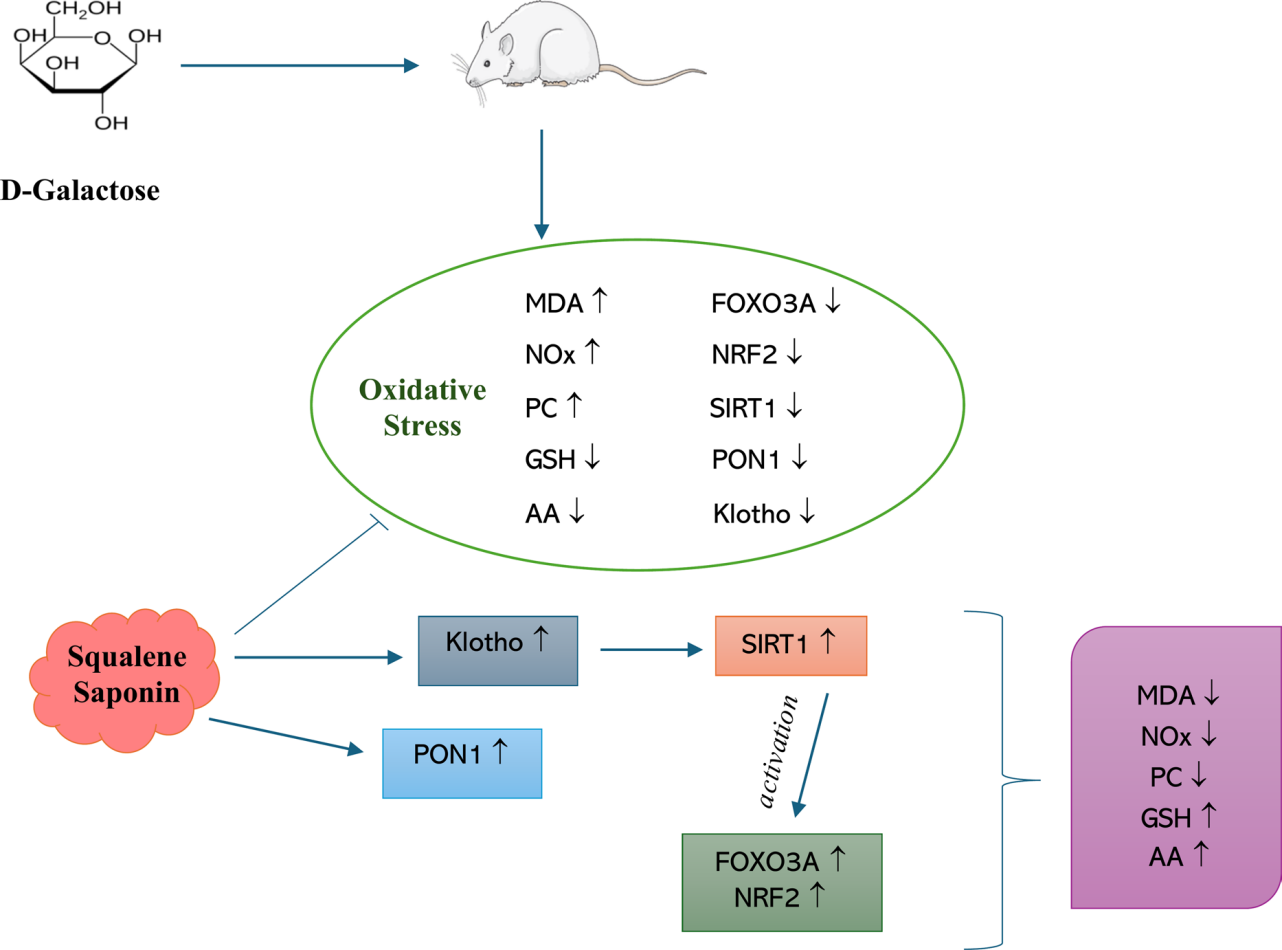

## Introduction

Compared with previous decades, the global population is currently aging at an unprecedented rate. Both the number of older individuals and their proportion within the total population are increasing across all countries, and it is projected that by 2050 nearly 80% of the elderly population will reside in low and middle-income countries (World Health Organization 2025). A similar demographic transition is evident in Turkey, where the proportion of individuals aged 65 years and older reached 10.6% of the total population as of 2024 (TUIK 2025). This rapid aging trend has rendered the elucidation of the biological mechanisms underlying age-related cognitive decline and neurodegenerative disorders a global public health priority.

Within molecular damage accumulation frameworks, oxidative stress–based theories provide a strong explanatory basis for the biochemical mechanisms of aging (Gladyshev 2014; Yang et al. 2024). The free radical theory of aging, originally proposed by Harman, suggested that the progressive accumulation of irreversible damage to cellular macromolecules caused by free radicals accelerates the aging process (Harman 1981). Currently, oxidative stress is recognized not merely as a damage-inducing mechanism but as a dynamic biological process involved in the regulation of cellular redox homeostasis through reactive oxygen species (ROS) and reactive nitrogen species (RNS). Under physiological conditions, ROS and RNS are natural byproducts of cellular metabolism and play functional roles in cell signaling, adaptive stress responses, and homeostatic regulatory mechanisms (Hong et al. 2024). When the production of these reactive species exceeds the capacity of endogenous antioxidant defense systems, redox homeostasis is disrupted, leading to oxidative stress. This imbalance contributes to the molecular basis of aging through the accumulation of damage in deoxyribonucleic acid (DNA), proteins, and lipids and represents a key trigger of cellular senescence (Cui et al. 2012; Jomova et al. 2023). Increased oxidative burden promotes permanent cell cycle arrest via activation of major regulatory signaling pathways, thereby limiting tissue regenerative capacity (Qin et al. 2025). In this context, redox-sensitive regulators involved in cellular antioxidant defense and metabolic adaptation play critical roles. Nuclear factor erythroid 2–related factor 2 (NRF2), Forkhead box O (FOXO) transcription factors, and members of the sirtuin (SIRT) family are recognized as molecular sensors responsive to oxidative stress and age-related redox imbalance. Age-associated alterations in the expression levels of these regulators have been linked to increased susceptibility to oxidative damage and reduced cellular adaptive capacity (Gupta et al. 2025; Hadinata et al. 2025).

The brain is particularly vulnerable to aging-related oxidative processes due to its high oxygen consumption and relatively limited antioxidant defense capacity. Age-associated mitochondrial dysfunction increased oxidative load, and chronic microglial activation lead to overlapping intermittent inflammatory responses, ultimately resulting in a state of persistent low-grade inflammation known as inflammaging (Ionescu-Tucker and Cotman 2021).

Experimental investigation of aging-related oxidative and inflammatory processes commonly employs the D-galactose (D-Gal)–induced aging model. Chronic D-Gal administration increases ROS and RNS production, promotes the accumulation of advanced glycation end products, and induces aging-like alterations, particularly in brain tissue, characterized by redox imbalance, mitochondrial damage, and neuronal apoptosis (Yanar et al. 2011; Azman and Zakaria 2019). These features render the D-Gal model a reliable and reproducible experimental system for evaluating mechanisms associated with oxidative stress, inflammation, and cellular senescence during aging.

The central role of oxidative stress in aging has intensified interest in antioxidant-based interventions. Squalene (SQ) is a natural terpenoid capable of efficiently scavenging lipid peroxidation–derived ROS and supporting cellular antioxidant defense pathways. In contrast, saponin (SP) enhances antioxidant enzyme systems and suppress inflammatory signaling pathways, thereby limiting the secondary consequences of oxidative damage (Li et al. 2014; Wang et al. 2016). Considering that a single antioxidant may be insufficient to effectively neutralize the full range of oxidant species, the combined use of SQ and SP may offer a complementary strategy that simultaneously targets ROS elimination and the regulation of inflammatory processes. From this perspective, this combination may have the potential to attenuate the temporal summation of intermittent inflammatory responses that occurs during the aging process.

In the present study, the effects of combined SQ and SP administration on molecular alterations related to oxidative stress and inflammation in brain tissue were investigated for the first time using a D-Gal-induced experimental aging model. The findings are expected to contribute to the development of holistic and mechanism based antioxidant strategies aimed at simultaneously modulating redox imbalance and inflammation during aging.

## Materials and methods

### Experimental animals and groups

Permission was obtained from the Gazi University Experimental Animals Ethics Committee (G.Ü. ET-23.028) for the studies, and all stages until the collection of tissue samples were carried out in the laboratory of Gazi University Laboratory Animal Breeding and Experimental Research Center. 48 male Sprague–Dawley rats (200–250 g) obtained from Gazi University Laboratory Animal Breeding and Experimental Research Center were used in this study. All animals received a standard laboratory chow ad libitum throughout the experimental period, and no dietary antioxidant or ascorbic acid (AA) supplementation was applied. During the experiment, the animals were housed in individual cages under a 12 h light/dark cycle and were randomly assigned to eight groups, as shown in Table 1.

**Table 1:** Experimental groups

Groups	Procedure
Control group	Saline was administered via oral gavage for 6 weeks (5 days per week) (*n* = 6)
D-Galactose group	D-Gal (300 mg/kg/day) was administered by intraperitoneal injection (IP) for 6 weeks (5 days per week) (*n* = 6)
D-Gal + SQ group	D-Gal (300 mg/kg/day) was administered by IP, and SQ (2.66 ml/kg/day) was administered via oral gavage for 6 weeks (5 days per week) (*n* = 6)
D-Gal + SP group	D-Gal (300 mg/kg/day) was administered by IP, and SP (100 mg/kg/day) was administered via oral gavage for 6 weeks (5 days per week) (*n* = 6)
D-Gal + combination group	D-Gal (300 mg/kg/day) was administered by IP, and SQ (2.66 ml/kg/day) and SP (100 mg/kg/day) were administered via oral gavage for 6 weeks (5 days per week) (n = 6)
Squalene group	SQ (2.66 ml/kg/day) was administered via oral gavage for 6 weeks (5 days per week) (n = 6)
Saponin group	SP (100 mg/kg/day) was administered via oral gavage for 6 weeks (5 days per week) (n = 6)
Combination group	SQ (2.66 ml/kg/day) and SP (100 mg/kg/day) were administered via oral gavage for 6 weeks (5 days per week) (n = 6)

### Experimental aging model and antioxidant regimen

Administrations were made to non-fasted rats in the morning (between 09.00 and 11.00) throughout the experimental period. This approach was chosen to minimize potential confounding effects of fasting-induced metabolic alterations, oxidative stress, and hormonal fluctuations on the evaluated biochemical parameters. All treatments were administered at the same time of day to ensure experimental standardization and reduce circadian variability. The first day on which the chemicals were administered was considered as day 0 of the experiment.

D-Gal used to create the aging model was adjusted to 300 mg/kg/day according to animal weight and was freshly prepared daily in 0.9% NaCl solution (Aydın et al. 2016; Saleh et al. 2019). Saponin (SAE0073, Sigma Aldrich, USA) to be used in the experiment was purchased commercially. It was freshly prepared in 0.9% NaCl solution and administered at a dose of 100 mg/kg/day (Abdel-Reheim et al. 2017). Squalene (S3626, Sigma Aldrich, USA) was purchased commercially in liquid form and administered at 2.66 ml/kg/day based on animal weight (Senthilkumar et al. 2006).

After the animals were weighed on a standard scale, D-Gal was administered IP at doses appropriate to their weight. SQ and SP were administered via oral gavage. D-Gal was administered at 300 mg/kg/day for 6 weeks, 5 days a week. To investigate the antioxidative effects, SQ, SP, and SQ + SP were administered to the 3rd, 4th, and 5th groups simultaneously with D-Gal administration. To present and compare the results accurately, SQ, SP, and SQ + SP were administered to the 6th, 7th, and 8th groups without D-Gal.

**Fig. 1:**
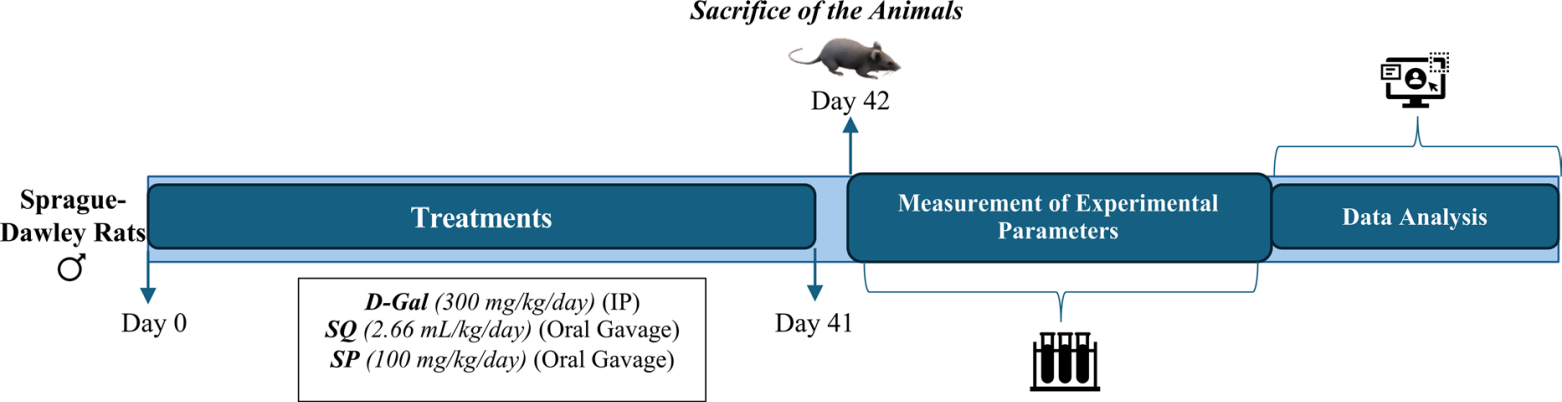
The experimental timeline schematic representation

The animals were sacrificed 24 h after the last administration by taking blood from their hearts under anesthesia (xylazine-ketamine). Following sacrifice, whole brain tissues were rapidly excised. No specific brain region was isolated, and homogenates were prepared from the entire brain tissue for all biochemical and molecular analyses. Biochemical parameters: MDA, glutathione (GSH), nitric oxide derivatives (NOx), AA, and protein carbonyls (PC) levels were measured using spectrophotometry. FOXO3A, NRF2, SIRT1, paraoxonase 1 (PON1), and Klotho levels in the brain tissues were determined by ELISA. Serum aspartate aminotransferase (AST) and alanine aminotransferase (ALT) levels were measured using the colorimetric method (Fig. 1).

### Analyses of redox biomarkers

Biochemical parameters were determined using routine methods at the Physiology Biochemistry Research Laboratory of the Department of Biology, Faculty of Science, Gazi University.

#### Malondialdehyde (MDA)

The content of lipid peroxidation products, thiobarbituric acid-reactive substances, in homogenates was measured. Tissue samples were weighed and homogenized in cold trichloroacetic acid (TCA) with a homogenizer. Thiobarbituric acid and butylated hydroxytoluene were then added to the supernatant. The optical densities of the samples were measured at 535 nm using a spectrophotometer. MDA levels in tissues were calculated as nmol/g of tissue (Casini et al. 1986).

#### Nitric oxide derivatives (NOx)

The NOx concentration in the tissues was determined using the Griess method. After the tissues were homogenized with sodium phosphate buffer, they were centrifuged at 3500 g for 15 min. An equal amount of vanadium(III) chloride was added to 200 μL of supernatant to reduce the nitrate in the medium to nitrite and incubated at 37 °C for 30 min. Sodium phosphate buffer and Griess I + II reagents were added, and after incubation for 10 min at 37 °C, the optical density of the samples was measured on a spectrophotometer at 540 nm (Miranda et al. 2001).

#### Glutathione (GSH)

The modified Ellman method was used to determine GSH levels in tissues. Tissue samples were homogenized in 0.15 M cold potassium chloride, and a mixture of meta- phosphoric acid/ethylenediaminetetraacetic acid/sodium chloride was added to 0.5 mL homogenate for deproteinization. After centrifugation at 4000 rpm for 20 min, 2 mL of 0.3 M disodium hydrogen phosphate and 0.2 mL of solution dithiobisnitrobenzoate (0.4 mg/ml 1% sodium citrate) were added to 0.5 mL of supernatant. The optical density of all samples was measured at 412 nm in a spectrophotometer. Tissue GSH levels were calculated in μmol per g of tissue (Aykac et al. 1985; Gürsoy et al. 2024).

#### Protein carbonyls (PC)

The PC levels were determined according to the method described by Reznick and Packer. Tissues were homogenized in 0.1 M phosphate buffer (pH 7.4) containing 1.17% KCl at a ratio of 1/10. To determine PC levels, two test tubes were taken, with and without 2,4-dinitrophenylhydrazine (DNPH), and homogenate (0.5 mL) was added to both tubes; 2 mL of DNPH was added to the tube with DNPH and 2 mL of HCl was added to the tube without DNPH. The tubes were vortexed every 15 min and incubated at room temperature for 1 h. 2.5 mL of 20% TCA was added to both tubes and the tubes were centrifuged at 12,000 rpm for 5 min, then the supernatant was discarded, and work continued with the pellet. After 2 mL of 10% TCA was added, the tubes were centrifuged at 12,000 rpm for 5 min and the supernatant was discarded. Ethanol/ethyl acetate (2 mL) was added to the tubes, then the tubes were centrifuged at 12,000 rpm for 5 min, and the supernatant was discarded. This process was repeated three times. 1 mL of guanidine HCl was added to the two tubes, and after vortexing for 10 min, the samples were incubated for a while to dissolve, and the absorbance values of the samples against guanidine HCl at 370 nm and 280 nm were recorded (Reznick and Packer 1994).

#### Ascorbic acid (AA)

Roe and Kuether’s method, modified by Berger, was used. Tissues were homogenized in an ice-cold perchloric acid / ethylenediaminetetraacetic acid mixture. The homogenate was centrifuged at 15,000 g (RCF) for 3 min at 4 °C. A standard AA solution was placed in one tube, the perchloric acid solution was placed in another tube for the blank, and the supernatant was placed in the tubes where the samples were prepared. Color reagent was added to each tube, vortexed, and incubated for 3 h at 37 °C. The temperature of the samples were brought to 0 °C and H2SO4 was added to each tube and mixed. The mixture was then incubated at room temperature for 30 min. The absorbance of the samples was measured in a spectrophotometer at 515 nm (Berger et al. 1989).

#### FOXO3A, NRF2, PON1, SIRT1, and Klotho

FOXO3A, NRF2, PON1, SIRT1, and Klotho levels in the tissues were determined using the ELISA method. The relevant tissues were weighed and homogenized with lysis buffer (1:50). The homogenates were then centrifuged at 3000 g for 5 min. Subsequently, the supernatants were analyzed using commercial ELISA kits (Cloud-Clone (Uscn), Rat ELISA Kits) through a service procurement process. Tissue levels of FOXO3A, NRF2, PON1, SIRT1, and Klotho were calculated and expressed as ng/mL.

#### Serum transaminase activities

AST and ALT levels in the serum were determined using a colorimetric method. Blood samples collected from the animals were centrifuged at 3500 rpm for 15 min, and the obtained serum samples were transferred into Eppendorf tubes, frozen in liquid nitrogen, and stored until analysis.

Serum AST and ALT analyses were performed through service procurement using commercial colorimetric assay kits (AST: OttoBC127; ALT: OttoBC128; Otto Scientific, Turkey) on a MINDRAY BS-400 automated biochemical analyzer. AST and ALT levels were calculated and expressed as U/L.

### Statistical analysis

All statistical analyses and graphical presentations were performed using GraphPad Prism version 8.4.2 (GraphPad Software, San Diego, CA, USA). Data are expressed as mean ± standard deviation (SD). Prior to inferential statistical analyses, the assumption of normality was evaluated by examining the distribution of residuals using the Shapiro–Wilk normality test. To further support the normality assumption, D’Agostino–Pearson omnibus and Anderson–Darling tests were also applied.

Differences among experimental groups were analyzed using one-way analysis of variance (one-way ANOVA). When a significant overall effect was observed, post hoc pairwise comparisons were conducted using Tukey’s multiple comparisons test. Differences were considered to be statistically significant at p < 0.05.

## Results

### MDA, NOx and PC levels

**Fig. 2:**
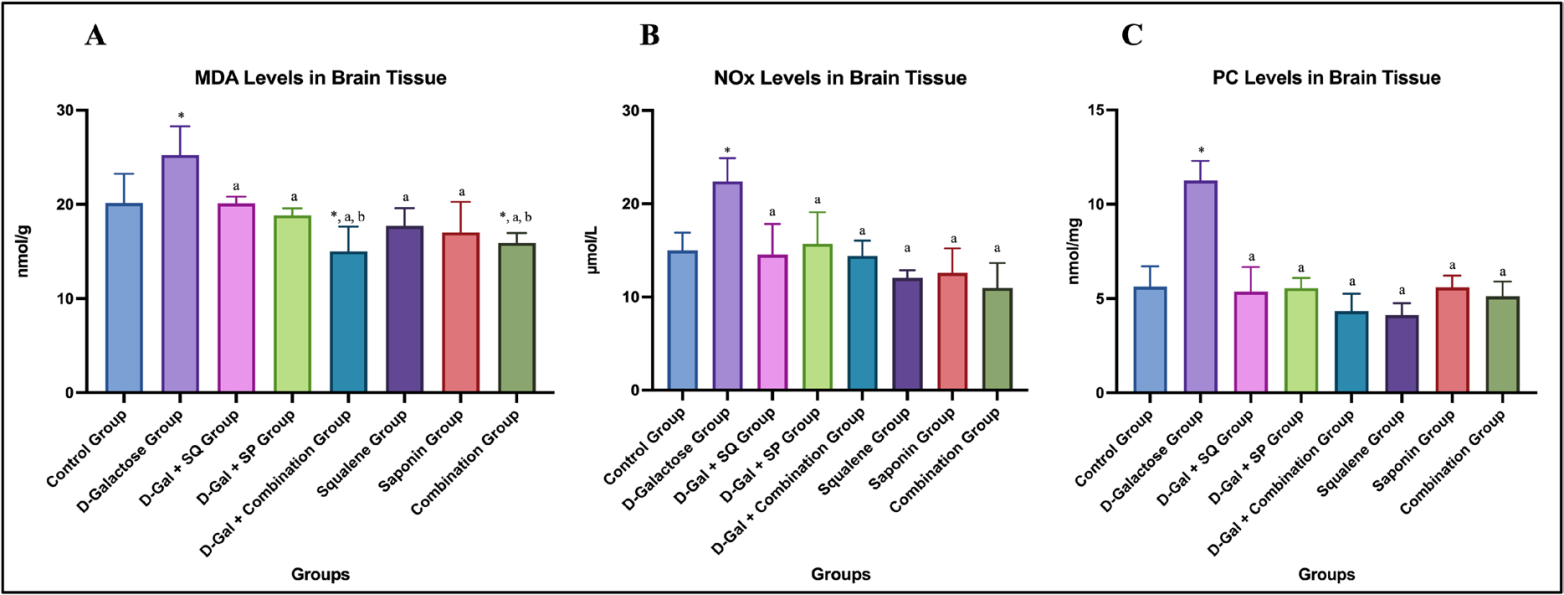
Graphs showing changes in brain tissue MDA (**A**), NOx (**B**), and PC (**C**) level in all groups. Values are expressed as mean ± SD..*: compared with the control group (*p* < 0.05), a: compared with the D-Galactose group (*p* < 0.05), **b**: compared with the D-Gal + SQ group (*p* < 0.05)***,*** c: compared with the D-Gal + SP group (*p* < 0.05), d: compared with the D-Gal + Combination group (p < 0.05), e: compared with the Squalene group (*p* < 0.05), f: compared with the Saponin group (*p* < 0.05)

In brain tissue, MDA levels, an indicator of lipid peroxidation, were significantly increased in the D-Galactose group compared to the control group (*p* < 0.05). In D-Gal-treated rats, administration of SQ, SP, or their combination significantly reduced MDA levels compared to the D-Galactose group (*p* < 0.05). Notably, MDA levels in the D-Gal + Combination group were significantly lower than those in the D-Gal + SQ group (*p* < 0.05). In antioxidant-treated groups without D-Gal exposure, a statistically significant difference was observed between the Combination group and the control group (*p* < 0.05). (Fig. 2A).

A statistically significant increase in NOx levels was observed in the D-Galactose group compared with the Control group (p < 0.05). In D-Gal–treated animals, administration of SQ, SP, or their combination significantly reduced NOx levels compared with the D-Galactose group (*p* < 0.05). (Fig. 2B).

PC levels, a marker of age-related protein oxidation in brain tissue, were significantly increased in the D-Galactose group compared with the control group (*p* < 0.05). Administration of SQ, SP, or their combination significantly reduced PC levels compared with the D-Galactose group (*p* < 0.05) (Fig. 2C).

### GSH and AA levels

**Fig. 3:**
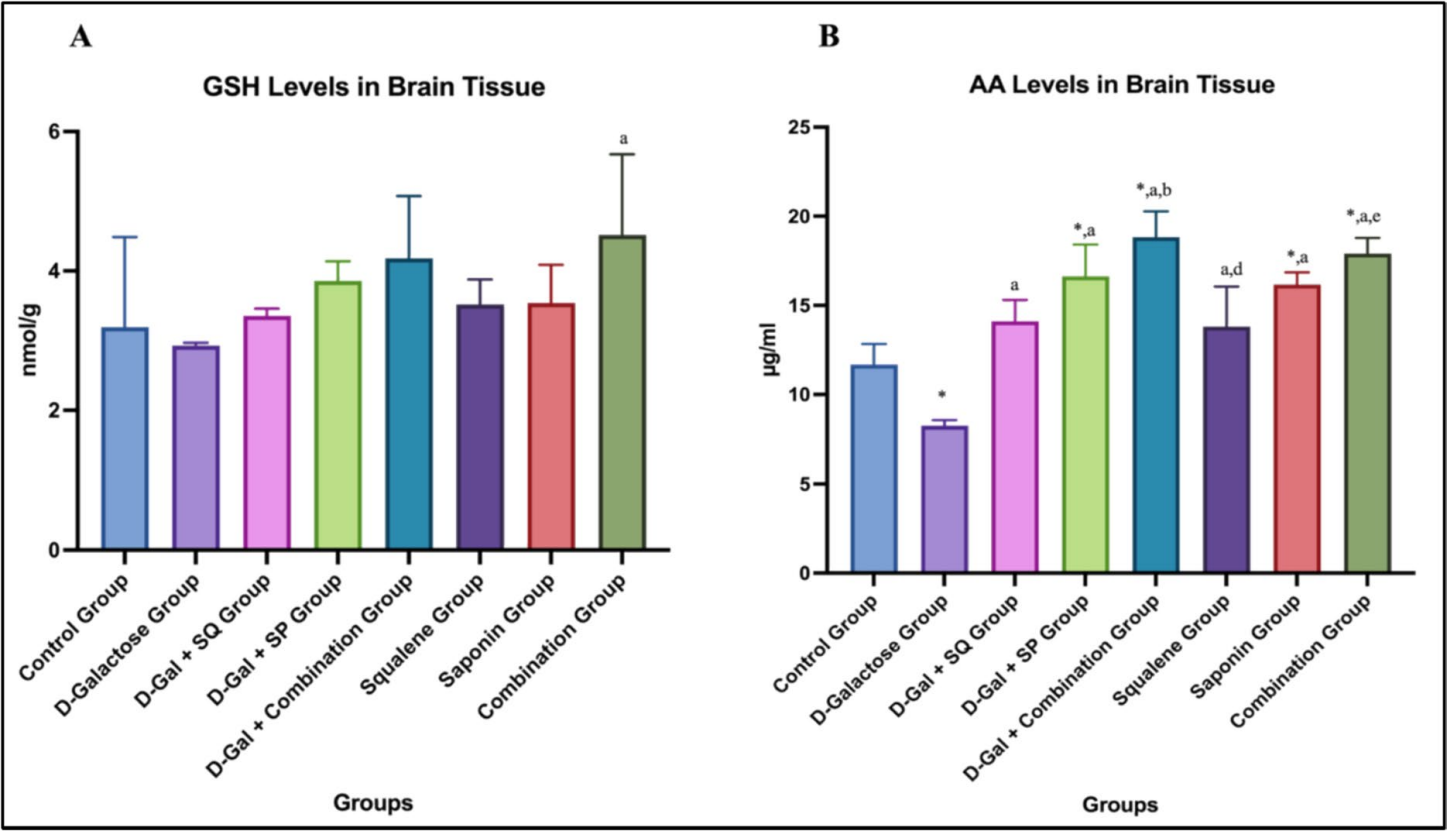
Graphs showing changes in brain tissue GSH (**A**) and AA (**B**) level in all groups. Values are expressed as mean ± SD..*: compared with the control group (*p* < 0.05), a: compared with the D-Galactose group (p < 0.05), **b**: compared with the D-Gal + SQ group (*p* < 0.05), **c**: compared with the D-Gal + SP group (*p* < 0.05), **d**: compared with the D-Gal + Combination group (*p* < 0.05), e: compared with the Squalene group (*p* < 0.05), f: compared with the Saponin group (*p* < 0.05)

Regarding GSH levels, an increase was observed in the D-Gal + SQ, D-Gal + SP, and D-Gal + Combination groups compared to the D-Galactose group; however, this difference was not statistically significant. In the SQ, SP, and Combination groups without D-Gal administration, GSH levels were numerically higher than those of the control group; however, no statistically significant differences were detected (Fig. 3A).

Brain tissue AA levels were significantly decreased in the D-Galactose group compared to the control group (*p* < 0.05). In D-Gal–treated animals, administration of SQ, SP, or their combination significantly increased AA levels compared to the D-Galactose group (*p* < 0.05). Notably, AA levels in the D-Gal + Combination group were significantly higher than those in the D-Gal + SQ group (p < 0.05). Among the groups not subjected to the aging model, AA levels were significantly higher in the Saponin and Combination groups compared to the control group (*p* < 0.05). In addition, a statistically significant difference was observed between the Combination and Squalene groups (*p* < 0.05) (Fig. 3B).

### SIRT1, FOXO3A, and NRF2 levels

**Fig. 4:**
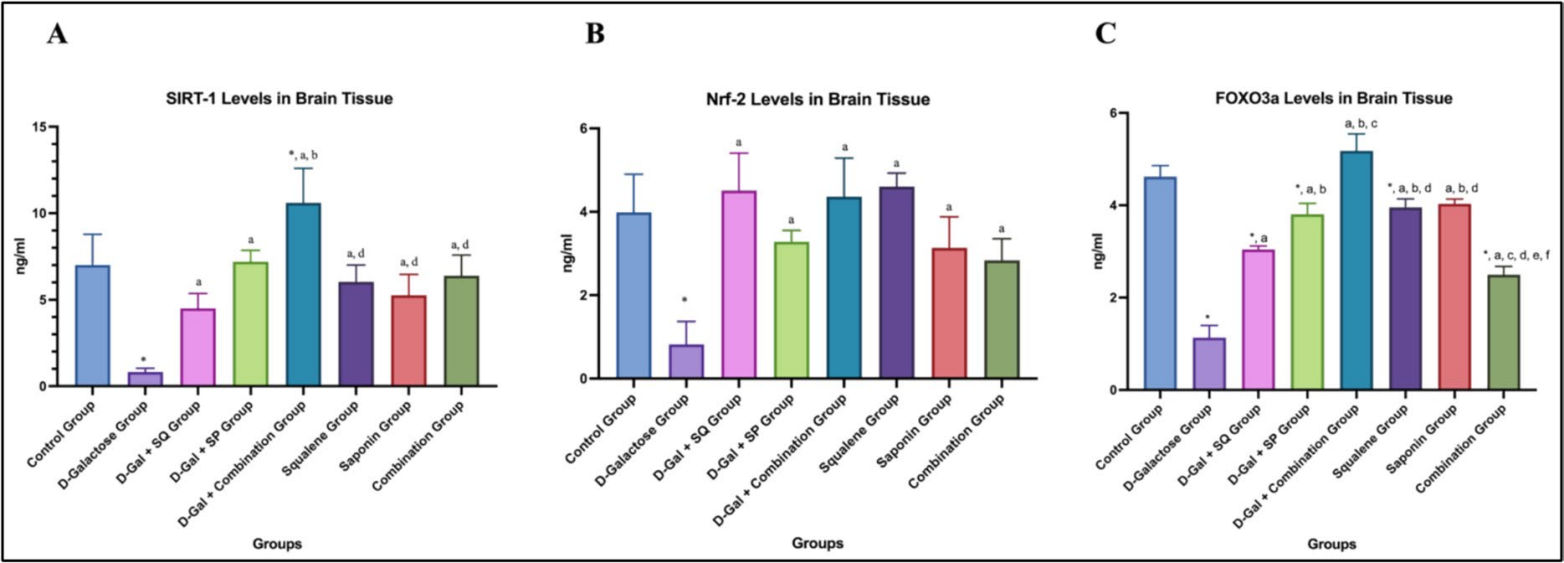
Graphs showing changes in brain tissue SIRT1 (**A**), NRF2 (**B**), and FOXO3A (**C**) level in all groups. Values are expressed as mean ± SD..*: compared with the control group (*p* < 0.05), a: compared with the D-Galactose group (*p* < 0.05)***,*** b: compared with the D-Gal + SQ group (*p* < 0.05)***,*** c: compared with the D-Gal + SP group (p < 0.05), d: compared with the D-Gal + Combination group (*p* < 0.05), e: compared with the Squalene group (*p* < 0.05), f: compared with the Saponin group (*p* < 0.05)

Brain SIRT1 levels were significantly reduced in the D-Galactose group compared to the control group (*p* < 0.05). In D-Gal–treated animals, administration of SQ, SP, or their combination significantly increased SIRT1 levels compared to the D-Galactose group (p < 0.05). Notably, SIRT1 levels were significantly higher in the D-Gal + Combination group compared with the D-Gal + SQ group (p < 0.05) (Fig. 4A).

When NRF2 levels were evaluated, a statistically significant difference was observed between the control and D-Galactose groups (p < 0.05). NRF2 levels were significantly increased in all antioxidant-treated groups compared to the D-Galactose group (*p* < 0.05) (Fig. 4B).

When FOXO3A levels in brain tissue were evaluated, a statistically significant difference was observed between the Control group and the D-Galactose group (*p* < 0.05). In D-Gal–treated animals, FOXO3A levels were significantly increased in all antioxidant-treated groups compared to the D-Galactose group (*p* < 0.05). Among these groups, the highest increase in FOXO3A levels was observed in the D-Gal + Combination group. FOXO3A levels in the D-Gal + Combination group were significantly higher than those in the D-Gal + SQ and D-Gal + SP groups (*p* < 0.05). In addition, a statistically significant difference was observed between the D-Gal + SP group and the D-Gal + SQ group (p < 0.05) (Fig. 4C).

### PON1 and Klotho levels

**Fig. 5:**
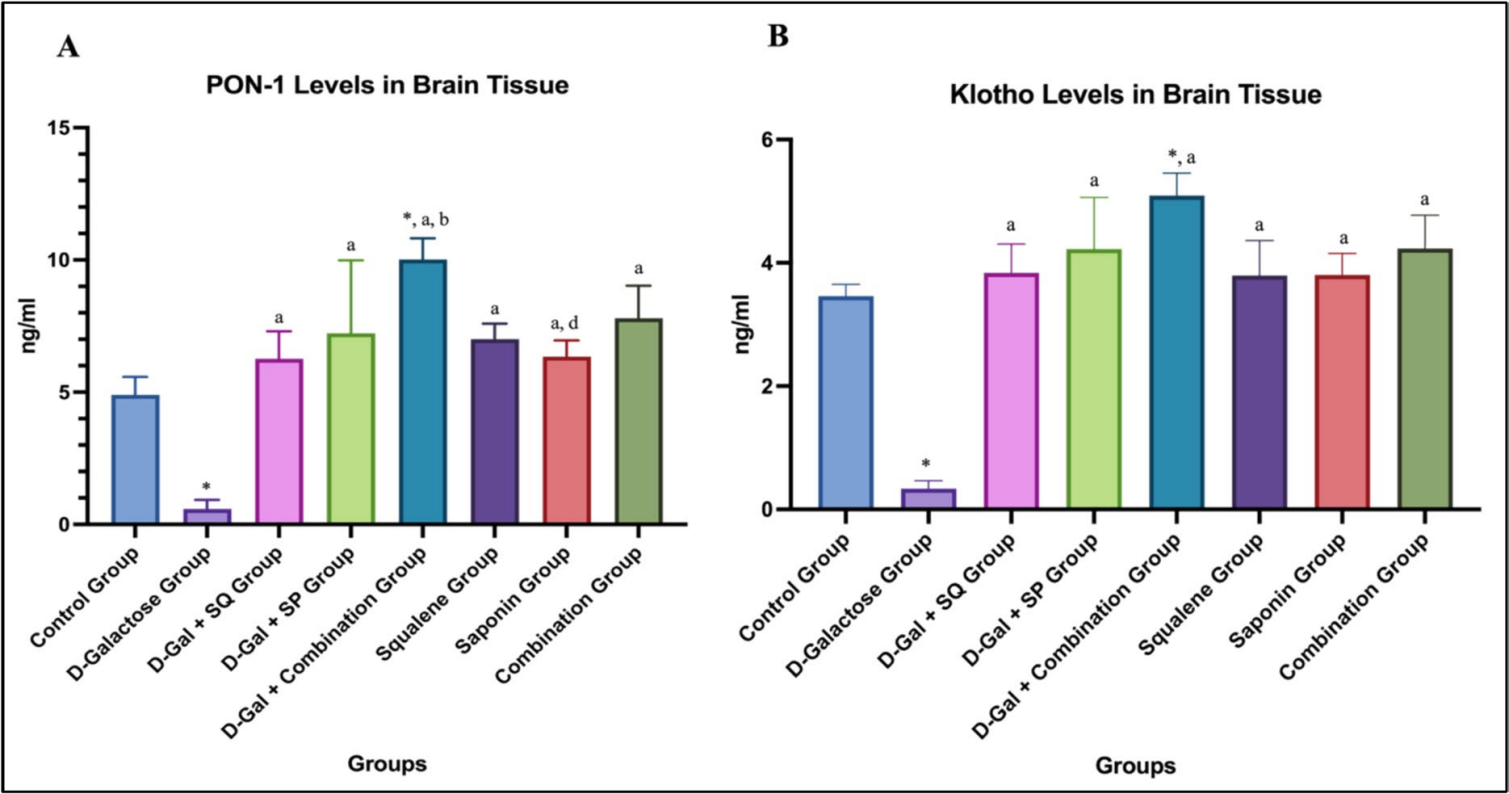
Graphs showing changes in brain tissue PON1 (**A**) and Klotho (**B**) level in all groups. Values are expressed as mean ± SD..*: compared with the control group (*p* < 0.05), **a**: compared with the D-Galactose group (*p* < 0.05), **b**: compared with the D-Gal + SQ group (*p* < 0.05), **c**: compared with the D-Gal + SP group (*p* < 0.05), **d**: compared with the D-Gal + Combination group (*p* < 0.05), e: compared with the Squalene group (*p* < 0.05), f: compared with the Saponin group (*p* < 0.05)

When PON1 levels in brain tissue were evaluated, a statistically significant difference was observed between the D-Galactose group and the Control group (p < 0.05). PON1 levels were significantly increased in all antioxidant-treated groups compared to the D-Galactose group (p < 0.05). In addition, a statistically significant difference was found between the D-Gal + Combination group and the D-Gal + SQ group (p < 0.05) (Fig. 5A).

When Klotho levels in brain tissue were evaluated, a statistically significant decrease was observed in the D-Galactose group compared to the Control group (p < 0.05). Klotho levels were significantly increased in all treatment groups compared to the D-Galactose group (p < 0.05). Notably, Klotho levels in the D-Gal + Combination group were significantly higher than those in the Control group (p < 0.05) (Fig. 5B).

### Serum ALT-AST levels

**Fig. 6:**
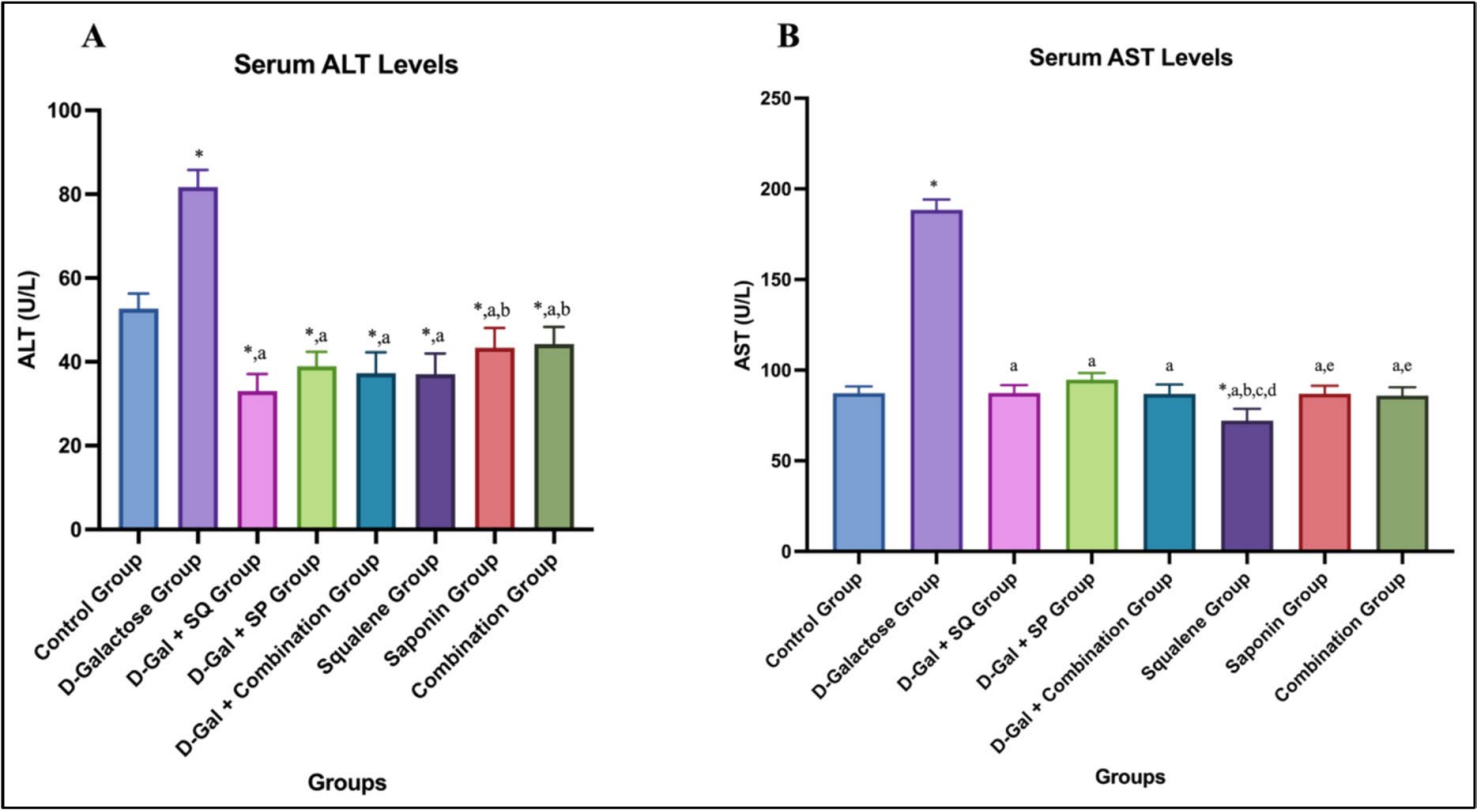
Graphs showing changes in serum ALT (**a**) and AST (**b**) level in all groups. Values are expressed as mean ± SD..*: compared with the control group (*p* < 0.05), a: compared with the D-Galactose group (*p* < 0.05)***,*** b: compared with the D-Gal + SQ group (*p* < 0.05), **c**: compared with the D-Gal + SP group (*p* < 0.05), **d**: compared with the D-Gal + Combination group (p < 0.05), **e**: compared with the Squalene group (p < 0.05), **f**: compared with the Saponin group (p < 0.05)

When serum ALT levels were evaluated, a statistically significant increase was observed in the D-Galactose group compared to the Control group (*p* < 0.05). ALT levels in the D-Galactose group were significantly higher than those in all antioxidant-treated groups (*p* < 0.05). Administration of SQ, SP, or their combination significantly reduced ALT levels compared to the D-Galactose group (*p* < 0.05). In groups without the aging model, ALT levels differed significantly from the control group (*p* < 0.05) (Fig. 6A).

When serum AST levels were evaluated, a statistically significant difference was observed between the Control group and the D-Galactose group (*p* < 0.05). In contrast, antioxidant-treated groups exhibited lower AST levels compared to the D-Galactose group (*p* < 0.05). Among these, the Squalene group showed the lowest AST levels, which were significantly lower than those observed in the Saponin and Combination groups (*p* < 0.05) (Fig. 6B).

## Discussion

Aging-related oxidative damage is particularly prominent in the brain due to its high lipid content, elevated oxygen consumption, and relatively limited antioxidant defense capacity (Cobley et al. 2018). Neuronal membranes are enriched in polyunsaturated fatty acids, making brain tissue especially vulnerable to lipid peroxidation–driven oxidative injury (Gai et al. 2025). In this context, the selection of lipid-associated antioxidant strategies represents a rational and tissue-specific approach for mitigating aging-related oxidative damage in the central nervous system.

Unlike hydrophilic antioxidants, lipid-soluble compounds may exert distinct advantages in lipid-rich tissues by directly interacting with membrane-associated oxidative processes and interrupting lipid peroxidation chain reactions (Niki 2014; Halliwell and Gutteridge 2015). However, studies that simultaneously evaluating multiple lipid-associated antioxidants with complementary biological properties in brain tissue remain limited. Therefore, investigating the combined effects of structurally and functionally distinct lipid-associated antioxidants in an experimental aging model provides an important contribution to the current understanding of antioxidant strategies targeting brain aging.

SQ exhibits high oral bioavailability owing to its lipophilic structure and is readily incorporated into lipid membranes, facilitating its effective distribution to peripheral tissues, including the brain (Reddy and Couvreur 2009; Kumar et al. 2023). In contrast, SP constitutes a structurally diverse class of glycosidic compounds whose oral bioavailability is generally limited; however, accumulating evidence indicates that SP can be metabolized by the intestinal microbiota into more lipophilic and biologically active derivatives, thereby enabling systemic and central effects. Notably, several SP-derived metabolites have been reported to exert neuroprotective, antioxidant, and anti-inflammatory effects either by crossing the blood–brain barrier or by preserving its integrity through indirect mechanisms (Sun et al. 2015).

Based on these complementary pharmacokinetic and biological properties, the selection of SQ and SP in the present study was motivated not by their isolated effects alone, but by the hypothesis that their combined administration may produce a cooperative or complementary protective effect, simultaneously targeting oxidative stress and inflammation associated with aging-related brain alterations.

The D-Gal-induced aging model is widely used to mimic accelerated aging and is characterized by increased lipid peroxidation, depletion of endogenous antioxidants, neuronal damage, and cognitive decline. Previous studies have demonstrated that natural antioxidants, including polyphenols, flavonoids, and terpenoids, attenuate D-Gal–induced oxidative and neuronal alterations (Al-babily and Tawfeeq 2020; Farajdokht et al. 2021; Zhong et al. 2024). Alpha-lipoic acid, for example, has been shown to improve mitochondrial function, reduce oxidative stress markers, enhance antioxidant defenses, and mitigate neuronal damage in experimental aging models (Erdogan et al. 2017).

D-Gal has been reported to promote oxidative stress and apoptosis through suppression of NRF2 and heme oxygenase-1 signaling, elevation of MDA levels, and reduction of glutathione peroxidase activity (GSH-Px) in the hippocampus (Rajendran 2024). The findings of the present study are consistent with these reports and confirm the successful induction of an aging-like phenotype characterized by redox imbalance.

Studies on *Panax japonicus*–derived SP have demonstrated increases in superoxide dismutase and GSH-Px activities and reductions in MDA levels (Wan et al. 2019). Similarly, SP administration in D-Gal–induced aging models attenuated brain MDA elevation and restored depleted GSH levels (Li et al. 2021). SQ has also been reported to reduce lipid hydroperoxide accumulation and enhance antioxidant enzyme activities, including superoxide dismutase, catalase, and GSH-Px, as well as to restore GSH levels in experimental models (Dhandapani et al. 2007; Ravi Kumar et al. 2016).

Consistent with these observations, the present study demonstrated that D-Gal administration significantly increased MDA levels in brain tissue, reflecting enhanced lipid peroxidation. Treatment with SQ, SP, or their combination significantly reduced MDA levels compared with the D-Galactose group. Within the D-Gal–treated groups, the combined SQ and SP regimen was associated with lower MDA levels than those observed in the single-compound treatment groups. These findings indicate a more pronounced protective outcome of the combined treatment under the experimental conditions applied.

Regulation of NOx levels plays an important role in protection against aging-related neuronal damage. The neuroprotective effects of SP have been partly attributed to suppression of NOx production and neuroinflammatory signaling (Kabir et al. 2023). SP administration normalized MDA and NOx levels in ischemia–reperfusion models (Zhang et al. 2014), while SQ was shown to suppress inducible nitric oxide synthase expression and limit NOx generation (Cárdeno et al. 2015). In the present study, D-Gal markedly increased NOx levels, whereas SQ, SP, and their combination significantly reduced NOx, supporting their effectiveness in modulating redox imbalance.

Increasing evidence indicates that ROS function not only as byproducts of metabolism but also as mediators of redox signaling. Both free radical and non-radical ROS regulate cellular signaling through reversible redox modifications of redox-sensitive proteins (Kumar et al. 2018; Atayik and Cakatay 2023). Dysregulation of ROS-mediated signaling disrupts physiological redox homeostasis and contributes to neurodegenerative processes.

Redox-sensitive transcriptional regulators, including NRF2, FOXO family members, and SIRT1, play critical roles in neuronal adaptation to oxidative stress. Excessive ROS disrupt these pathways and accelerate neurodegeneration (Holubiec et al. 2023). In this study, D-Gal-induced senescence was associated with elevated oxidative stress markers and altered levels of NRF2, FOXO3A, and SIRT1, indicating disruption of redox-sensitive signaling networks. SQ and SP treatments partially normalized these alterations, suggesting modulation of redox-responsive regulatory pathways rather than direct evidence of transcriptional activation.

Previous studies have shown that SP restores FOXO3A and SIRT1 expression in aging models (Wan et al. 2019), and dysregulation of FOXO3A has been implicated in neurodegenerative diseases (Desplats et al. 2012; Wang et al. 2020). In line with these findings, FOXO3A levels were reduced in the D-Gal group and increased following antioxidant treatments, with higher levels observed in the combined treatment group.

NRF2 is a central regulator of antioxidant defense and functionally interacts with FOXO3A. Although NRF2 transcriptional activity was not directly assessed in the present study, changes in total NRF2 protein levels were evaluated as indicators of redox-responsive molecular alterations. D-Gal reduced NRF2 levels, whereas antioxidant treatments increased its expression, with SQ contributing notably to this effect.

SIRT1 plays a central role in aging and neuronal survival and is known to decline with age (Li et al. 2014; Wang et al. 2015). In the present study, D-Gal reduced SIRT1 levels, while antioxidant treatments restored its expression, accompanied by improvements in FOXO3A and NRF2-associated responses.

PON1 activity declines with aging and is associated with increased oxidative stress (Loued et al. 2013). Expression of PON1 and PON3 in brain tissue suggests a role in cerebral redox regulation (Salazar et al. 2021). In this study, D-Gal reduced PON1 levels, whereas antioxidant treatments—particularly combined SQ and SP—were associated with increased PON1 expression. SQ has been reported to enhance high density lipoprotein (HDL)–PON1 interactions (Gabás-Rivera et al. 2014), and reduced PON1 activity has been linked to neurodegenerative diseases (Mota et al. 2019; Arslan et al. 2016).

Klotho regulates aging-related pathways and oxidative stress responses, and reduced expression is associated with neurodegeneration (Shaker et al. 2021; Kanbay et al. 2024). D-Gal significantly reduced Klotho levels, whereas antioxidant treatments increased its expression, with higher levels observed in the combined treatment group. Klotho is known to interact with SIRT1 and redox-sensitive transcription factors, including NRF2 and FOXO3A (de Freitas Silva et al. 2018; Brunet et al. 2004).

Overall, the findings of this study are consistent with previous reports implicating the Klotho–SIRT1–FOXO3A–NRF2 signaling network in the regulation of oxidative stress and aging-related processes. SQ and SP appear to support endogenous defense mechanisms in the aging brain primarily through modulation of redox-sensitive regulatory systems.

AST and ALT are widely used indicators of liver function. AST is present in multiple tissues, whereas ALT is predominantly localized in hepatocytes and is considered more specific for hepatocellular injury (Li et al. 2022). Oxidative stress is a key mechanism underlying age-related liver dysfunction, and elevations in serum ALT and AST are closely associated with ROS-induced lipid peroxidation and mitochondrial impairment. Accordingly, the increased transaminase levels observed following D-Gal administration likely reflect ROS-mediated hepatocellular damage, while their reduction by antioxidant treatments suggests attenuation of oxidative liver injury.

Recent studies have demonstrated hepatoprotective effects of SP supplementation in models of oxidative liver damage, including reductions in serum AST and ALT via antioxidant and anti-inflammatory mechanisms (Abdel-Reheim et al. 2022; Qiu et al. 2023).

This study demonstrated that D-Gal administration disrupted redox balance in brain tissue, evidenced by changes in the levels of age-related key regulators such as FOXO3A, NRF2, SIRT1, PON1, and Klotho, increased oxidative damage markers, and decreased endogenous antioxidants. Antioxidant treatments partially reversed these changes, and co-administration of SQ and SP led to improvements in various parameters compared to individual treatments. However, given the limited number of studies directly linking these compounds to age-related neurodegenerative conditions, further research is needed to clarify their potential roles in age-related brain dysfunction.

## Conclusion

As the global elderly population continues to increase, the identification of tissue-specific strategies that support healthy aging has become an important research priority. The present study demonstrates that D-Gal administration induces aging-like alterations in brain tissue, characterized by increased oxidative stress, disruption of redox balance, and alterations in key molecular regulators associated with aging.

Given the lipid-rich composition of brain tissue and its vulnerability to lipid peroxidation, the use of lipid-associated antioxidant compounds represents a rational and biologically relevant approach. In this context, administration of SQ and SP was associated with attenuation of oxidative damage markers, partial restoration of endogenous antioxidant components, and modulation of redox-sensitive regulators, including FOXO3A, NRF2, SIRT1, PON1, and Klotho.

Aging is accompanied by recurrent and overlapping inflammatory responses that contribute to persistent low-grade inflammation. Within the applied experimental framework, the combined administration of SQ and SP was associated with favorable modulation of multiple oxidative and molecular parameters, suggesting a potential influence on redox–inflammation dynamics under aging-related conditions.

Overall, the findings indicate that SQ and SP exert protective effects against D-Gal–induced oxidative and molecular alterations in this experimental aging model. The combined administration of these compounds was associated with favorable modulation of multiple oxidative and aging-related parameters within the applied experimental framework. Further studies employing factorial experimental designs and in-depth molecular analyses are warranted to clarify the pathways involved, evaluate dose-dependent effects, and assess the relevance of these findings to age-associated disorders and other tissues in the context of healthy aging.

## Data Availability

The datasets generated and analyzed during the current study are available from the corresponding author upon reasonable request.

## References

[CR1] Abdel-Reheim MA, Messiha BAS, Abo-Saif AA (2017) Quillaja saponaria bark saponin protects wistar rats against ferrous sulphate-induced oxidative and inflammatory liver damage. Pharm Biol 55:1972–1983. 10.1080/13880209.2017.1345950 28728456 10.1080/13880209.2017.1345950 PMC6130630

[CR2] Abdel-Reheim MA, Ashour AA, Khattab MA, Gaafar AGA (2022) Quillaja saponaria bark saponin attenuates methotrexate induced hepatic oxidative stress, inflammation and associated liver injury in rats. J Appl Pharm Sci 12:129–141. 10.7324/JAPS.2022.120510

[CR3] Al-babily E, Tawfeeq F (2020) Effect of melatonin on some biochemical parameters in d-galactose induced aging in rats. Rafidain J Sci 29:39–47. 10.33899/rjs.2020.164473

[CR4] Arslan A, Tüzün FA, Arslan H et al (2016) The relationship between serum paraoxonase levels and carotid atherosclerotic plaque formation in Alzheimer’s patients. Neurol Neurochir Pol 50:403–409. 10.1016/j.pjnns.2016.07.002 27546893 10.1016/j.pjnns.2016.07.002

[CR5] Atayik MC, Çakatay U (2023) Redox signaling and modulation in ageing. Biogerontology 24(5):603–608. 10.1007/s10522-023-10055-w 37535201 10.1007/s10522-023-10055-w

[CR6] Aydın AF, Çoban J, Doğan-Ekici I et al (2016) Carnosine and taurine treatments diminished brain oxidative stress and apoptosis in d-galactose aging model. Metab Brain Dis 31:337–345. 10.1007/s11011-015-9755-0 26518192 10.1007/s11011-015-9755-0

[CR7] Aykac G, Uysal M, Yalcin AS et al (1985) The effect of chronic ethanol ingestion on hepatic lipid peroxide, glutathione, glutathione peroxidase and glutathione transferase in rats. Toxicology 36:71–76. 10.1016/0300-483X(85)90008-3 4040665 10.1016/0300-483x(85)90008-3

[CR8] Azman KF, Zakaria R (2019) D-galactose-induced accelerated aging model: an overview. Biogerontology 20:763–782. 10.1007/s10522-019-09837-y 31538262 10.1007/s10522-019-09837-y

[CR9] Berger J, Shepard D, Morrow F, Taylor A (1989) Relationship between dietary intake and tissue levels of reduced and total vitamin C in the nonscorbutic guinea pig. J Nutr 119:734–740. 10.1093/jn/119.5.734 2723822 10.1093/jn/119.5.734

[CR10] Brunet A, Sweeney LB, Sturgill JF et al (2004) Stress-dependent regulation of foxo transcription factors by the sirt1 deacetylase. Science 303:2011–2015. 10.1126/science.1094637 14976264 10.1126/science.1094637

[CR11] Cárdeno A, Aparicio-Soto M, Montserrat-de la Paz S et al (2015) Squalene targets pro- and anti-inflammatory mediators and pathways to modulate over-activation of neutrophils, monocytes and macrophages. J Funct Foods 14:779–790. 10.1016/j.jff.2015.03.009

[CR12] Casini AF, Ferrali M, Pompella A et al (1986) Lipid peroxidation and cellular damage in extrahepatic tissues of bromobenzene-intoxicated mice. Am J Pathol 123:520 3717304 PMC1888269

[CR13] Cobley JN, Fiorello ML, Bailey DM (2018) 13 reasons why the brain is susceptible to oxidative stress. Redox Biol 15:490–503. 10.1016/j.redox.2018.01.008 29413961 10.1016/j.redox.2018.01.008 PMC5881419

[CR14] Cui H, Kong Y, Zhang H (2012) Oxidative stress, mitochondrial dysfunction, and aging. J Signal Transduct 2012:646354. 10.1155/2012/646354 21977319 10.1155/2012/646354 PMC3184498

[CR15] de Freitas Silva M, Pruccoli L, Morroni F et al (2018) The Keap1/Nrf2-ARE pathway as a pharmacological target for chalcones. Molecules 23:1803. 10.3390/molecules23071803 30037040 10.3390/molecules23071803 PMC6100069

[CR16] Desplats P, Patel P, Kosberg K et al (2012) Combined exposure to Maneb and Paraquat alters transcriptional regulation of neurogenesis-related genes in mice models of Parkinsons disease. Mol Neurodegener 7:49. 10.1186/1750-1326-7-49 23017109 10.1186/1750-1326-7-49 PMC3502617

[CR17] Dhandapani N, Ganesan B, Anandan R et al (2007) Synergistic effects of squalene and polyunsaturated fatty acid concentrate on lipid peroxidation and antioxidant status in isoprenaline-induced myocardial infarction in rats. Afr J Biotechnol 6

[CR18] Erdoğan ME, Aydın S, Yanar K, Mengi M, Kansu AD, Cebe T, Belce A, Çelikten M, Çakatay U (2017) The effects of lipoic acid on redox status in brain regions and systemic circulation in streptozotocin-induced sporadic Alzheimer’s disease model. Metab Brain Dis 32(4):1017–1031. 10.1007/s11011-017-9983-6 28299625 10.1007/s11011-017-9983-6

[CR19] Farajdokht F, Sadigh-Eteghad S, Mahmoudi J (2021) d-galactose-induced aging and brain mitochondria. Assessments. The Neuroscience of Aging. Academic Press, Treatments and Modeling in Aging and Neurological Disease, pp 471–480

[CR20] Gabás-Rivera C, Barranquero C, Martínez-Beamonte R et al (2014) Dietary squalene increases high density lipoprotein-cholesterol and paraoxonase 1 and decreases oxidative stress in mice. PLoS ONE. 10.1371/journal.pone.0104224 25117703 10.1371/journal.pone.0104224 PMC4130590

[CR21] Gai K, Bu F, Qi S (2025) The physiology of brain cells: interactions between free radicals and lipid peroxidation. Cell Physiol Ann. 10.5772/intechopen.115620

[CR22] Gladyshev VN (2014) The free radical theory of aging is dead. Long live the damage theory! Antioxid Redox Signal 20(4):727–731. 10.1089/ars.2013.5228 24159899 10.1089/ars.2013.5228 PMC3901353

[CR23] Gupta S, Afzal M, Agrawal N, Almalki WH, Rana M, Gangola S et al (2025) Harnessing the FOXO-SIRT1 axis: insights into cellular stress, metabolism, and aging. Biogerontology 26(2):65. 10.1007/s10522-025-10207-0 40011269 10.1007/s10522-025-10207-0

[CR24] Gürsoy EN, Balabanli KB, Tuğcu Demiröz FN, Coşkun Cevher Ş (2024) The cumulative effect of ellagic acid and carnosic acid attenuates oxidative events during diabetic wound healing: in different applications and on different days. Turk J Biol 48(6):364–378. 10.55730/1300-0152.2712 39758844 10.55730/1300-0152.2712 PMC11698191

[CR25] Hadinata E, Harbuwono DS, Soegondo S et al (2025) Marine nutraceuticals as a source of SIRT1 and NRF2 activators for diabetes and aging-related metabolic disorders. Diabetol Metab Syndr 17:1–31 41094589 10.1186/s13098-025-01981-5 PMC12529833

[CR26] Halliwell B, Gutteridge JM (2015) Free radicals in biology and medicine. Oxford University Press

[CR27] Harman D (1981) The aging process. Proc Natl Acad Sci U S A 78:7124–7128. 10.1073/pnas.78.11.7124 6947277 10.1073/pnas.78.11.7124 PMC349208

[CR28] Holubiec MI, Gellert M, Hanschmann EM (2023) Editorial: redox regulation and signaling in neurodegenerative diseases. Front Aging Neurosci 15:1135303. 10.3389/fnagi.2023.1135303 36733721 10.3389/fnagi.2023.1135303 PMC9887324

[CR29] Hong Y, Boiti A, Vallone D, Foulkes NS (2024) Reactive oxygen species signaling and oxidative stress: transcriptional regulation and evolution. Antioxidants 13:312. 10.3390/antiox13030312 38539845 10.3390/antiox13030312 PMC10967436

[CR30] Ionescu-Tucker A, Cotman CW (2021) Emerging roles of oxidative stress in brain aging and Alzheimer’s disease. Neurobiol Aging 107:86–95. 10.1016/j.neurobiolaging.2021.07.014 34416493 10.1016/j.neurobiolaging.2021.07.014

[CR31] Jomova K, Raptova R, Alomar SY, Alwasel SH, Nepovimova E, Kuca K, Valko M (2023) Reactive oxygen species, toxicity, oxidative stress, and antioxidants: chronic diseases and aging. Arch Toxicol 97(10):2499–2574. 10.1007/s00204-023-03562-9 37597078 10.1007/s00204-023-03562-9 PMC10475008

[CR32] Kabir MT, Shah MA, Alothaim AAS et al (2023) Neuroprotective effects of saponins on neurodegenerative diseases. In: Khan H, Aschner M, Mirzaei H (eds) Phytonutrients and Neurological Disorders: Therapeutic and Toxicological Aspects. Academic Press, London, pp 259–282

[CR33] Kanbay M, Copur S, Ozbek L et al (2024) Klotho: a potential therapeutic target in aging and neurodegeneration beyond chronic kidney disease-a comprehensive review from the ERA CKD-MBD working group. Clin Kidney J. 10.1093/ckj/sfad276 38213484 10.1093/ckj/sfad276 PMC10783249

[CR34] Kumar A, Yegla B, Foster TC (2018) Redox signaling in neurotransmission and cognition during aging. Antioxid Redox Signal 28(18):1724–1745. 10.1089/ars.2017.7111 28467718 10.1089/ars.2017.7111 PMC5962336

[CR35] Kumar LR, Tejpal CS, Anas KK, Chatterjee NS, Anandan R, Mathew S, Ravishankar CN (2023) Squalene: bioactivity, extraction, encapsulation, and future perspectives. In Marine Antioxidants, Academic Press, pp 409–419. 10.1016/B978-0-323-95086-2.00038-2

[CR36] Li YN, Guo Y, Xi MM et al (2014) Saponins from aralia taibaiensis attenuate d-galactose-induced aging in rats by activating FOXO3a and Nrf2 pathways. Oxid Med Cell Longev. 10.1155/2014/320513 24669284 10.1155/2014/320513 PMC3942195

[CR37] Li H, Zhai B, Sun J et al (2021) Antioxidant, anti-aging and organ protective effects of total saponins from Aralia taibaiensis. Drug des Devel Ther 15:4025–4042. 10.2147/DDDT.S330222 34594101 10.2147/DDDT.S330222 PMC8476322

[CR38] Li W, Yue L, Sun L, Xiao S (2022) An increased aspartate to alanine aminotransferase ratio is associated with a higher risk of cognitive impairment. Front Med (lausanne). 10.3389/fmed.2022.780174 35463002 10.3389/fmed.2022.780174 PMC9021637

[CR39] Loued S, Berrougui H, Componova P et al (2013) Extra-virgin olive oil consumption reduces the age-related decrease in HDL and Paraoxonase 1 anti-inflammatory activities. Br J Nutr 110:1272–1284. 10.1017/S0007114513000482 23510814 10.1017/S0007114513000482

[CR40] Miranda KM, Espey MG, Wink DA (2001) A rapid, simple spectrophotometric method for simultaneous detection of nitrate and nitrite. Nitric Oxide 5:62–71. 10.1006/niox.2000.0319 11178938 10.1006/niox.2000.0319

[CR41] Mota A, Hemati-Dinarvand M, Taheraghdam AA et al (2019) Association of paraoxonse1 (PON1) genotypes with the activity of PON1 in patients with Ptarkinson’s disease. Acta Neurol Taiwan 28:66–74 32002976

[CR42] Niki E (2014) Role of vitamin E as a lipid-soluble peroxyl radical scavenger: in vitro and in vivo evidence. Free Radic Biol Med 66:3–12. 10.1016/j.freeradbiomed.2013.03.022 23557727 10.1016/j.freeradbiomed.2013.03.022

[CR43] Park C, Cha HJ, Song KS, Kim HS, Bang E, Lee H, Jin CY, Kim GY, Choi YH (2023) Nrf2-mediated activation of HO-1 is required in the blocking effect of compound K, a ginseng saponin metabolite, against oxidative stress damage in ARPE-19 human retinal pigment epithelial cells. J Ginseng Res 47(2):311–318. 10.1016/j.jgr.2022.09.007 36926611 10.1016/j.jgr.2022.09.007 PMC10014180

[CR44] Qin Y, Liu H, Wu H (2025) Cellular senescence in health, disease, and lens aging. Pharmaceuticals (Basel) 18(2):244. 10.3390/ph18020244 40006057 10.3390/ph18020244 PMC11859104

[CR45] Qiu L, Feng R, Wu QS et al (2023) Total saponins from Panax japonicus attenuate acute alcoholic liver oxidative stress and hepatosteatosis by p62-related Nrf2 pathway and AMPK-ACC/PPARα axis in vivo and in vitro. J Ethnopharmacol. 10.1016/j.jep.2023.116785 37321425 10.1016/j.jep.2023.116785

[CR46] Rajendran P et al (2024) Geraniol attenuates oxidative stress and neuroinflammation-mediated cognitive impairment in D galactose-induced mouse aging model. Aging Albany NY 16:5000–5026 38517361 10.18632/aging.205677 PMC11006477

[CR47] Ravi Kumar S, Narayan B, Sawada Y et al (2016) Combined effect of astaxanthin and squalene on oxidative stress in vivo. Mol Cell Biochem 417:57–65. 10.1007/s11010-016-2713-2 27188184 10.1007/s11010-016-2713-2

[CR48] Reddy LH, Couvreur P (2009) Squalene: a natural triterpene for use in disease management and therapy. Adv Drug Deliv Rev 61(15):1412–1426. 10.1016/j.addr.2009.09.005 19804806 10.1016/j.addr.2009.09.005

[CR49] Reznick AZ, Packer L (1994) Oxidative damage to proteins: spectrophotometric method for carbonyl assay. Methods Enzymol 233:357–363. 10.1016/S0076-6879(94)33041-7 8015470 10.1016/s0076-6879(94)33041-7

[CR50] Salazar JG, Marsillach J, Reverte I et al (2021) Paraoxonase-1 and-3 protein expression in the brain of the tg2576 mouse model of alzheimer’s disease. Antioxidants 10:339. 10.3390/antiox10030339 33668379 10.3390/antiox10030339 PMC7996151

[CR51] Saleh DO, Mansour DF, Hashad IM, Bakeer RM (2019) Effects of sulforaphane on D-galactose-induced liver aging in rats: role of keap-1/nrf-2 pathway. Eur J Pharmacol 855:40–49. 10.1016/j.ejphar.2019.04.043 31039346 10.1016/j.ejphar.2019.04.043

[CR52] Senthilkumar S, Yogeeta SK, Subashini R, Devaki T (2006) Attenuation of cyclophosphamide induced toxicity by squalene in experimental rats. Chem Biol Interact 160:252–260. 10.1016/j.cbi.2006.02.004 16554041 10.1016/j.cbi.2006.02.004

[CR53] Shaker MR, Aguado J, Chaggar HK, Wolvetang EJ (2021) Klotho inhibits neuronal senescence in human brain organoids. NPJ Aging Mech Dis 7:18. 10.1038/s41514-021-00070-x 34341344 10.1038/s41514-021-00070-x PMC8329278

[CR54] Sun A, Xu X, Lin J, Cui X, Xu R (2015) Neuroprotection by saponins. Phytother Res 29(2):187–200 25408503 10.1002/ptr.5246

[CR55] TUIK (2025) İstatistiklerle Yaşlılar, (2024) Türkiye İstatistik Kurumu. In: Retrieved from https://data.tuik.gov.tr/Bulten/Index?p=Elderly-Statistics-2024-54079

[CR56] Wan J, Wang R, Zhou Z et al (2019) Saponins of *panax japonicus* confer neuroprotection against brain aging through mitochondrial related oxidative stress and autophagy in rats. Curr Pharm Biotechnol 21:667–680. 10.2174/1389201021666191216114815 10.2174/1389201021666191216114815 31840608

[CR57] Wang T, Di G, Yang L et al (2015) Saponins from *Panax japonicus* attenuate D-galactose-induced cognitive impairment through its anti-oxidative and anti-apoptotic effects in rats. J Pharm Pharmacol 67:1284–1296. 10.1111/jphp.12413 25892055 10.1111/jphp.12413

[CR58] Wang Y, Ren T, Zheng L et al (2016) Astragalus saponins inhibits lipopolysaccharide-induced inflammation in mouse macrophages. Am J Chin Med 44:579–593. 10.1142/S0192415X16500324 27109155 10.1142/S0192415X16500324

[CR59] Wang Y, Lin Y, Wang L et al (2020) TREM2 ameliorates neuroinflammatory response and cognitive impairment via PI3K/AKT/FoxO3a signaling pathway in Alzheimer’s disease mice. Aging 12:20862. 10.18632/aging.104104 33065553 10.18632/aging.104104 PMC7655179

[CR60] World Health Organization (2025) Ageing and health. Available at: https://www.who.int/news-room/fact-sheets/detail/ageing-and-health. Accessed: 13 Jan 2026.

[CR61] Yanar K, Aydin S, Çakatay U et al (2011) Protein and DNA oxidation in different anatomic regions of rat brain in a mimetic ageing model. Basic Clin Pharmacol Toxicol 109:423–433. 10.1111/j.1742-7843.2011.00756.x 21733122 10.1111/j.1742-7843.2011.00756.x

[CR62] Yang J, Luo J, Tian X, Zhao Y, Li Y, Wu X (2024) Progress in understanding oxidative stress, aging, and aging-related diseases. Antioxidants 13:394. 10.3390/antiox13040394 38671842 10.3390/antiox13040394 PMC11047596

[CR63] Zhang X, Chen L, Dang X et al (2014) Neuroprotective effects of total steroid saponins on cerebral ischemia injuries in an animal model of focal ischemia/reperfusion. Planta Med 80:637–644. 10.1055/s-0034-1368584 24963614 10.1055/s-0034-1368584 PMC4083247

[CR64] Zhong R, Shen L, Fan Y, Luo Q et al (2024) Anti-aging mechanism and effect of treatment with raw and wine-steamed Polygonatum sibiricum on D-galactose-induced aging in mice by inhibiting oxidative stress and modulating gut microbiota. Front Pharmacol 15:1335786 38774211 10.3389/fphar.2024.1335786 PMC11106437

